# Supplementary material for: DNA methylation and transcriptional trajectories during human development and reprogramming of isogenic pluripotent stem cells
Source: Nat Commun. 2017 Oct 13;8:908. doi: 10.1038/s41467-017-01077-3 (PMC5640655; doi:10.1038/s41467-017-01077-3)
Supplement: Supplementary file 1 — Supplementary Information [file 41467_2017_1077_MOESM1_ESM.pdf]

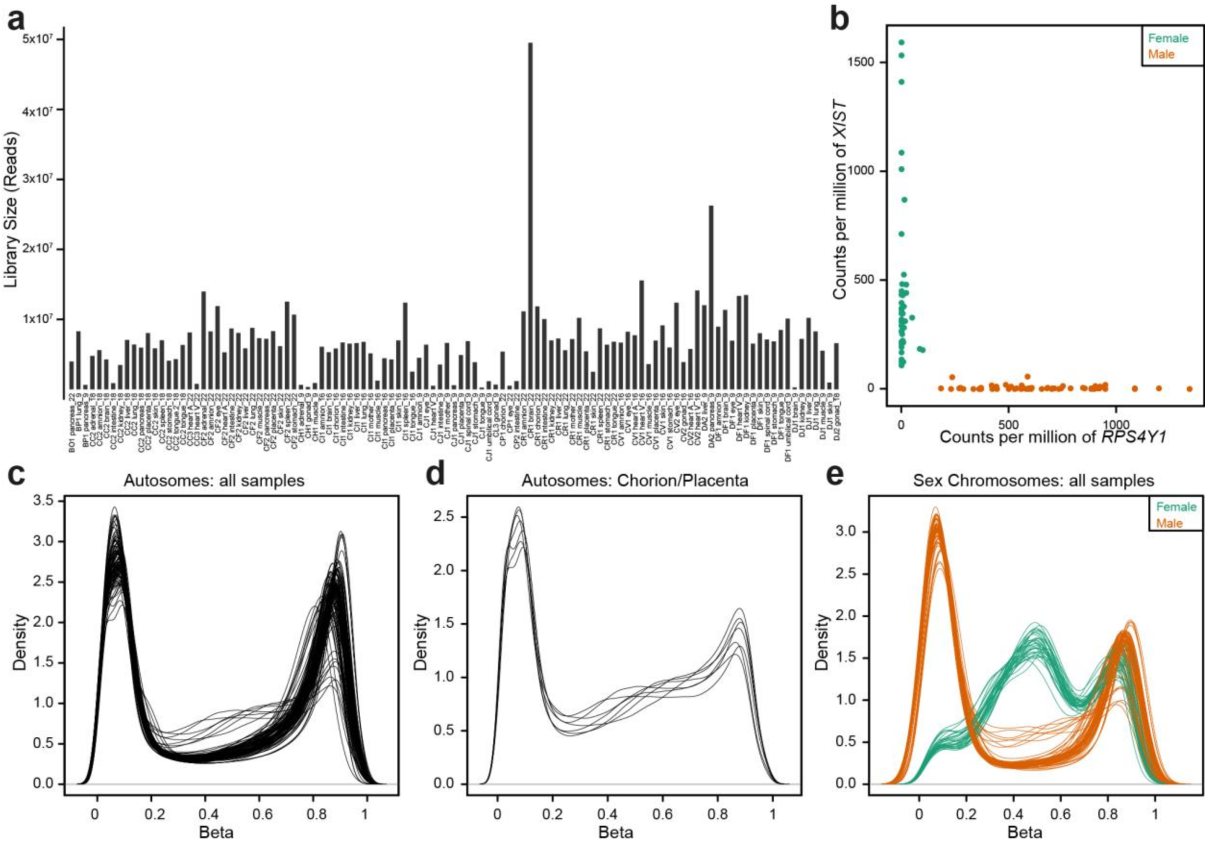

Supplementary Figure 1. Quality control of transcriptome and DNA methylome data. (a) Library sizes of the 111 NGS transcriptional profiles. **(b)** Expression levels (counts per million, CPM) of the female gene *XIST* and the male gene *RPS4Y1*. **(c-d)** Density plots of the beta values (450K DNA methylation) of the autosomes of all samples **(c)** and only of the chorion and placenta **(d)**. **(e)** Density plot of the beta values (450K DNA methylation) of the sex chromosomes of all samples separated by sex (male and female).

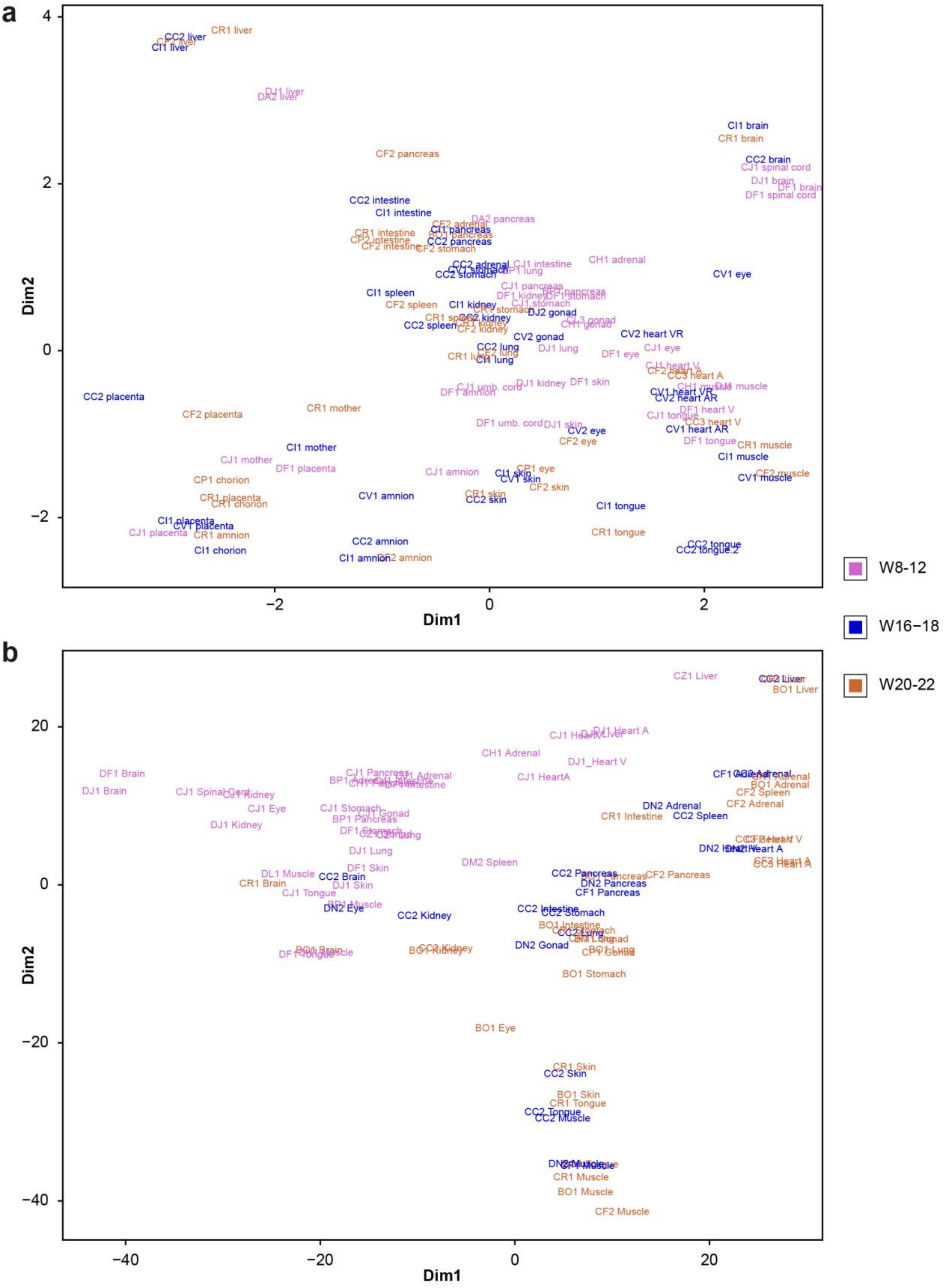
a
b
Dim1
Dim2
W8-12
W16–18
W20-22
CR1 liver
CC2 liver
CI1 liver
CI1 brain
CR1 brain
CC2 brain
CJ1 spinal cord
DJ1 brain
DF1 spinal cord
CF2 pancreas
CC2 intestine
CI1 intestine
DA2 pancreas
CR1 intestine
CC2 pancreas
CH1 adrenal
CV1 eye
CI1 spleen
CF2 spleen
CI1 kidney
CC2 spleen
DJ2 gonad
CV2 heart VR
CV2 gonad
DJ1 lung
DF1 eye
CJ1 eye
CC3 heart A
CV2 heart AR
DJ1 kidney
DF1 skin
CC2 placenta
CF2 placenta
CR1 mother
CJ1 tongue
DF1 heart V
CC3 heart V
CV1 heart AR
DF1 umb. cord
CV2 eye
CF2 eye
DF1 tongue
CR1 muscle
CI1 mother
CI1 muscle
CJ1 mother
DF1 placenta
CJ1 amnion
CP1 chorion
CF2 skin
CV1 amnion
CC2 skin
CI1 tongue
CR1 tongue
CJ1 placenta
CR1 amnion
CC2 amnion
CI1 chorion
CC2 tongue
CZ1 Liver
BO1 Liver
DJ1 Heart A
CH1 Adrenal
DJ1_Heart V
CJ1 HeartA
DF1 Brain
DJ1 Brain
CJ1 Eye
DJ1 Kidney
BP1 Pancreas
DN2 Adrenal
CF2 Spleen
CF2 Adrenal
CR1 Intestine
CC2 Spleen
DJ1 Lung
DM2 Spleen
DF1 Skin
DL1 Muscle
CR1 Brain
CC2 Brain
DJ1 Skin
CJ1 Tongue
CC2 Pancreas
CF2 Pancreas
DN2 Pancreas
CF1 Pancreas
CC2 Kidney
DN2 Gonad
BO1 Stomach
BO1 Eye
CC2 Skin
BO1 Skin
CR1 Tongue
CR1 Muscle
BO1 Muscle
CF2 Muscle

Supplementary Figure 2. Multidimensional scaling. (a-b) Multidimensional scaling (Euclidian distance) of the 111 transcriptional profiles (including extraembryonic samples) **(a)** and the 87 DNA methylation profiles of the embryonic samples only (excluding extraembryonic samples) **(b)**. Each sample is named as “embryo ID code_tissue name” and the color code depicts the gestational age group (W8-12 in pink; W16-18 in blue, W20-22 in orange).

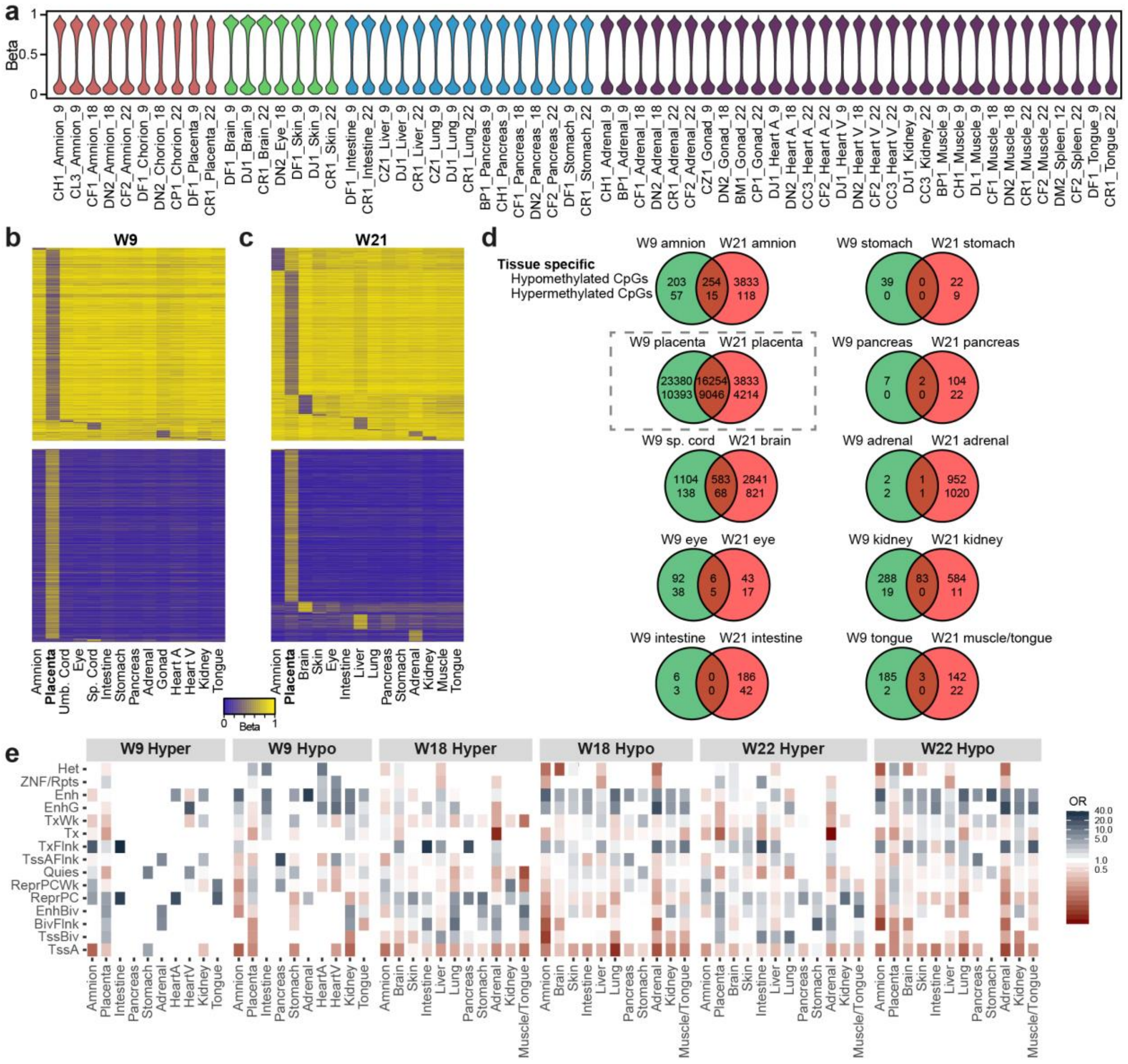

Supplementary Figure 3. DNA methylation signatures during human fetal development. (a) Violin plot depicting the distribution of the DNA methylation levels of the remaining samples that were excluded from the three isogenic sets shown in Fig. 3a. **(b-c)** Heatmaps displayed in Fig. 3b,c including the probes of the placenta. **(d)** Venn diagrams showing the overlap between W9 and W21 of tissue-specific hypomethylated CpGs (top) and of hypermethylated (bottom). The dashed-box highlights the large number of tissue specific CpGs in the placenta. **(e)** Enrichment of the identified hyper- and hypomethylated CpGs in the chromatin state segmentations for fetal adrenal, amnion, fetal heart atrium and ventricle, fetal intestine, fetal kidney, adult liver, fetal lung, fetal muscle, adult pancreas, placenta, adult fibroblasts (skin), and fetal stomach given as odds ratio (OR).

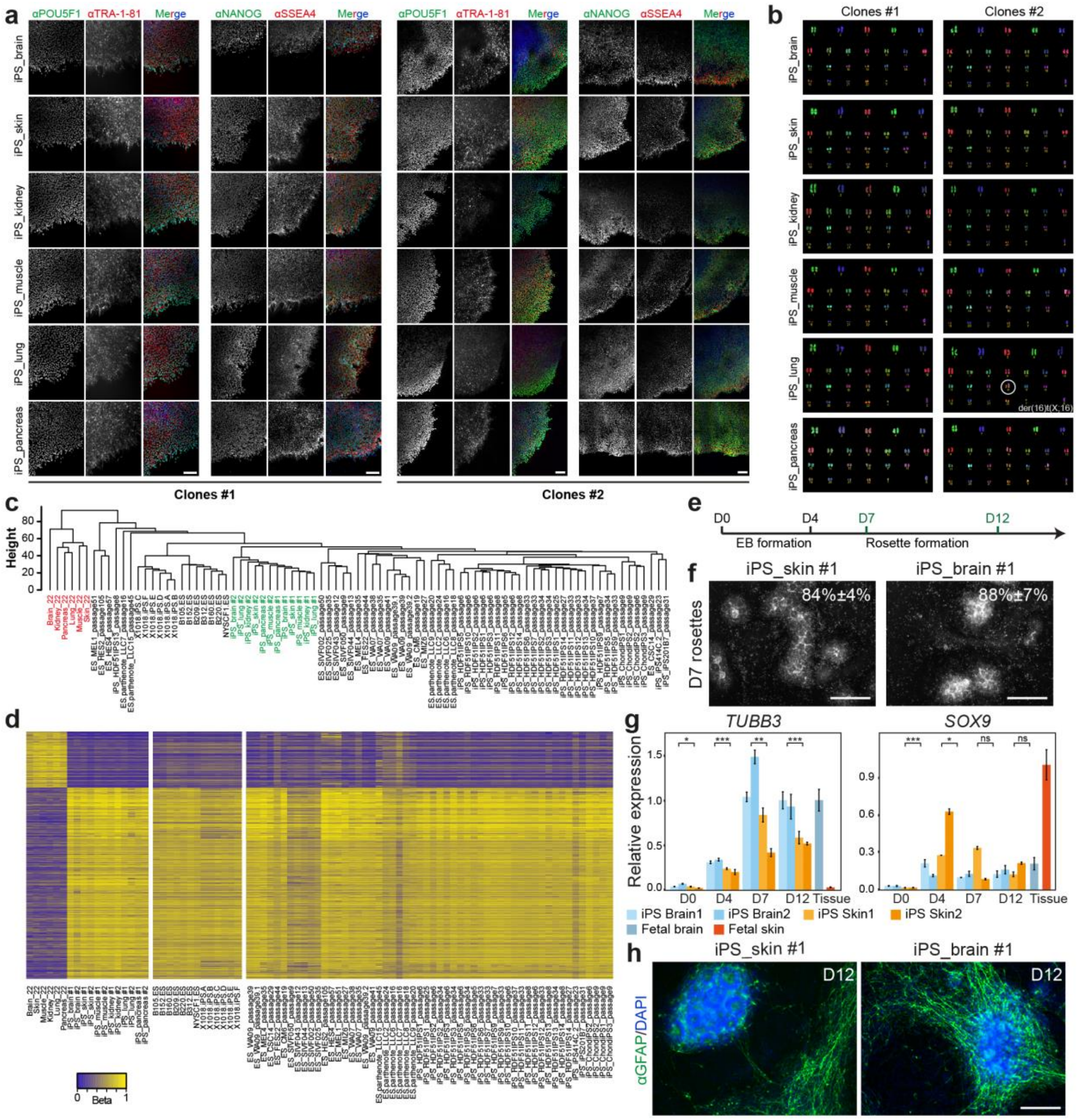

Supplementary Figure 4. Isogenic sets of human iPSCs. (a) Single and merged channels of the immunofluorescence of each of the 12 iPSC clones for POU5F1 and TRA-1-81, and NANOG and SSEA4. Scale bars 100 µm. **(b)** Karyograms of the 12 iPSC clones using COBRA. **(c)** Hierarchical clustering of the 12 iPSC clones (in green) and their tissues of origin (in red) together with two external 450K datasets (Johannesson et al., 2014; Nazor et al., 2012). **(d)** DNA methylation levels of two external datasets regarding the hiPSC-specific hypomethylated (2852 CpGs) and hypermethylated (8772 CpGs) CpG depicted in Fig. 4f. **(e)** Cartoon depicting the neural induction protocol followed and time points of analysis (in

green). **(f)** Dark field picture of embryoid bodies containing rosettes present in culture after 7 days of neural differentiation. Scale bars: 500 µm. **(g)** Relative expression of *TUBB3* and *SOX9* in hiPSCs and fetal brain and skin. Each bar represents mean ± standard deviation of technical triplicates. Significant P values between brain- and skin-hiPSCs at each day are indicated by ns, not significant; * ($p < 0.05$); ** ($p < 0.01$); and *** ($p < 0.001$); Student's t-test (two-tailed). **(h)** Immunofluorescence for the neural marker GFAP after 12 (D12) days of neural differentiation. Scale bars: 200 µm.
