# Supplementary material for: DNA methylation and transcriptional trajectories during human development and reprogramming of isogenic pluripotent stem cells
Source: Nat Commun. 2017 Oct 13;8:908. doi: 10.1038/s41467-017-01077-3 (PMC5640655; doi:10.1038/s41467-017-01077-3)
Supplement: Supplementary file 3 — Description of Additional Supplementary Files [file 41467_2017_1077_MOESM3_ESM.pdf]

Description of Additional Supplementary Files

File Name: Supplementary Data 1
Description: Illumina 450K samples and matching RNA sequencing.

File Name: Supplementary Data 2
Description: Differentially expressed genes (unique) between W8 and W22.

File Name: Supplementary Data 3
Description: Delta beta of the probes associated with up- and downregulated (organ-specific) genes.

File Name: Supplementary Data 4
Description: Nearest loci associated with hyper- and hypomethylated organspecific DMRs.

File Name: Supplementary Data 5
Description: Hyper- and hypomethylated probes in hiPSCs.

File Name: Supplementary Data 6
Description: Gene ontology analysis of hyper- and hypomethylated probes in hiPSCs.
